# Health Effects of Long-Term Exposure to Ambient PM_2.5_ in Asia-Pacific: a Systematic Review of Cohort Studies

**DOI:** 10.1007/s40572-022-00344-w

**Published:** 2022-03-16

**Authors:** Zhengyu Yang, Rahini Mahendran, Pei Yu, Rongbin Xu, Wenhua Yu, Sugeesha Godellawattage, Shanshan Li, Yuming Guo

**Affiliations:** Department of Epidemiology and Preventive Medicine, School of Public Health and Preventive Medicine, Monash University, VIC 3004 Melbourne, Australia

**Keywords:** Long-term exposure, Particulate matter, Health effect, Asia-Pacific, Systematic review

## Abstract

**Abstract:**

**Purpose of Review:**

Health effects of long-term exposure to ambient PM_2.5_ vary with regions, and 75% of the deaths attributable to PM_2.5_ were estimated in Asia-Pacific in 2017. This systematic review aims to summarize the existing evidence from cohort studies on health effects of long-term exposure to ambient PM_2.5_ in Asia-Pacific.

**Recent Findings:**

In Asia-Pacific, 60 cohort studies were conducted in Australia, Mainland China, Hong Kong, Taiwan, and South Korea. They consistently supported associations of long-term exposure to PM_2.5_ with increased all-cause/non-accidental and cardiovascular mortality as well as with incidence of cardiovascular diseases, type 2 diabetes mellitus, kidney diseases, and chronic obstructive pulmonary disease. Evidence for other health effects was limited. Inequalities were identified in PM_2.5_-health associations.

**Summary:**

To optimize air pollution control and public health prevention, further studies need to assess the health effects of long-term PM_2.5_ exposure in understudied regions, the health effects of long-term PM_2.5_ exposure on mortality and risk of type 2 diabetes mellitus, renal diseases, dementia and lung cancer, and inequalities in PM_2.5_-health associations. Study design, especially exposure assessment methods, should be improved.

**Supplementary Information:**

The online version contains supplementary material available at 10.1007/s40572-022-00344-w.

## Introduction

Particulate matter with a diameter of less than 2.5 μm (PM_2.5_) has been associated with cardiovascular diseases (CVD) [1•, 2•], respiratory diseases [3, 4], neurological diseases [5, 6, 7•], cancers [8••], and subsequent deaths [9, 10, 11••]. It was estimated responsible for four million deaths and 142 million disability-adjusted life years (DALY) worldwide in 2017 [12] and has been regarded as a primary health hazard by most countries.

Long-term (≥1 year) exposure to PM_2.5_ can lead to cumulative or chronic health effects, and the health effects may vary across regions because of differences in population characteristics, as well as sources and components of PM_2.5_ [13, 14, 15•, 16]. Therefore, some reviews have been conducted to synthesize health effects of long-term exposure to PM_2.5_ to provide evidence to local governments to formulate national or region-specific air pollution control and public health intervention policies [8••, 17].

Although concentrations of PM_2.5_ decreased in some regions (e.g., East China, South Central China, Southeast Asia, and Australasia) during 2000–2017 [18], PM_2.5_ is still a major health hazard in Asia-Pacific (i.e., East Asia, South Asia, Southeast Asia, and Oceania), where 75% of the estimated global deaths attributable to PM_2.5_ occurred in 2017 [12], and the hazard of ambient PM_2.5_ (main sources: traffic, industry, energy production and agriculture) is increasingly surpassing the hazard of indoor PM_2.5_ (main sources: combustion of solid fuel and biomass) in Asia-Pacific as a result of urbanization and industrialization [12, 19•]. However, there is a lack of systematic reviews for the health effects of long-term ambient PM_2.5_ exposure in Asia-Pacific. Most of the systematic reviews conducted previously have only covered selective health effects in Asia-Pacific, which were not able to fully describe the health impacts of long-term exposure to ambient PM_2.5_ for decision-makers and public health practitioners in Asia-Pacific [13, 20].

To fill this gap, we conducted a systematic review to answer the following Population, Exposure, Comparison, Outcome, Study Design (PECOS) question: what were the health effects of long-term exposure to ambient PM_2.5_ in any population, including subgroups of susceptible adults and children in Asia-Pacific, according to cohort studies published during 2000–2020?

## Materials and methods

This systematic review was conducted following the Preferred Reporting Items for Systematic Reviews and Meta-Analyses (PRISMA). The PRISMA checklist was presented in Section 1 of Supplementary Material. The protocol of this systematic review had been registered on PROSPERO (https://www.crd.york.ac.uk/prospero/) with an identification number of CRD42021254095.

### Search strategy

We performed a systematic literature search across three major databases: Medline, Embase, and Web of Science (WoS), with a time restriction of 2000–2020. We considered literatures in all languages. However, Chinese literature databases were not included because literature searches in major Chinese literature databases (i.e., China National Knowledge Infrastructure and VIP Information) consistently demonstrated there were only <200 Chinese journal articles about health effects of PM_2.5_, and none of them were cohort studies (Section 2 of Supplementary Material). We generated extensive search keywords for PM_2.5_: “air adj1 pollut*”, “air quality”, “atmospher* adj1 pollut*”, “air adj1 contamina*”, “particulate matter*”, and “fine particle*”. The study design was limited to cohort studies by keeping records with relevant keywords (i.e., “cohort stud*”, “cohort analys*”, “follow up stud*”, “longitudinal stud*”, “prospective stud”, and “retrospective stud*”) and excluding records with irrelevant subject headings or keywords (e.g., “cross over studies”, “cross-sectional studies”, “case-control studies”, and “clinical trial”.) Only cohort studies were considered because they provided the highest level of evidence for prognostic research questions [21]. To restrict the study area to Asia-Pacific, we excluded the records with subject headings such as “africa”, “europe”, and “americas” in Medline and Embase and refined the records using the country/region filter in WoS. The full search terms varied slightly across databases and were shown in Section 3 of Supplementary Material. The latest search was conducted on July 6, 2021. The bibliographies of relevant reviews identified through the literature search were also considered. To avoid excluding eligible literatures by mistake, the searches were not restricted by PM_2.5_ exposure term (long/short), although only long-term effects would be evaluated.

### Screening and selection

Two researchers (ZY, RM) independently screened the records that were identified in the literature search and the bibliographies of relevant reviews according to their titles and abstracts utilizing Covidence (https://www.covidence.org/). We excluded (1) reviews, meta-analyses, response letters, and conference abstracts; (2) clinical trials, in vitro studies (e.g., experiments on cells), cross-sectional studies, case-control studies, and ecological studies; (3) studies for non-human species; (4) studies for indoor or occupational exposure to PM_2.5_; (5) studies with an indirect exposure measurement (e.g., distance to major roads or pollution sources); (6) studies with a short- or medium-term (<1 year) exposure period; (7) studies conducted exclusively in areas other than Asia-Pacific. Inconsistencies between the two researchers were resolved by discussion. Unresolved inconsistencies and uncertainties were left to the full text screening.

To resolve the inconsistencies and uncertainties, and to assess the eligibility of the remaining records, two researchers (ZY, RM) independently read the full texts with referring to predetermined inclusion criteria: (1) original articles; (2) with a cohort study design; (3) human studies; (4) exposure was ambient PM_2.5_; (5) exposure period was long-term (≥1 year); (6) conducted within Asia-Pacific (multiregional studies were also eligible if any health effect within Asia-Pacific was reported). For multiple articles of the same outcome, population, and cohort, only the most recently published one was included. Consistencies between researchers were reached by discussion. Disagreements on eligibility were resolved by consulting a senior researcher (RX).

### Data extraction and quality assessment

Two researchers (ZY, SG) performed data extraction and quality assessment for all included studies independently. For each study, we extracted authors, publication year, country/region, study period, study population, number of events, sample size, sex, age (range was extracted if both mean ± standard deviation [SD] and median [IQR] were not reported), exposure measurement (method, spatial resolution, mean exposure, and exposure window), outcome of interest (definition and ascertainment method), covariate adjustment, and health effect estimates. Only single-pollutant models were considered unless just multi-pollutant models were available. Categorical analyses (e.g., relative health effects of high exposure compared to low exposure) were considered when health effect estimates per increment in exposure were not available. Data from sensitivity analyses were not extracted. When multiple models (usually incrementally adjusted models) were presented for the same outcome variable, we extracted data from the designated main model (usually shown in the abstract), or, if a main model was not clearly designated, from the most adjusted model. The Newcastle Ottawa Scale (NOS) for cohort studies was used to assess the quality of included studies [22]. Consensus were achieved by discussion or consulting senior researchers (PY, WY).

### Data analysis

Due to the small numbers of studies for most health outcomes and great heterogeneities in the study populations, exposure assessment methods, and covariate adjustments, we only conducted qualitative syntheses, after excluding low-quality studies (NOS score <5). Extracted health effect estimates were grouped according to the International Classification of Diseases 10^th^ version (ICD-10) based on their definition described in the articles and were standardized as estimated changes per 10 μg/m^3^ increase in long-term exposure to ambient PM_2.5_ if applicable. The standardized health effect estimates were further synthesized using forest plots if they were measured as relative risk changes (i.e., risk ratio [RR], odds ratio, and hazard ratio [HR]). As for health effect estimates extracted from categorical analyses, which could not be transformed to linear effect estimates on a continuous scale (i.e., changes per 10 μg/m^3^ increase in exposure) or health effect estimates not measured as relative risk changes, we reported their directions and significance with negative (when upper limit of 95% confidence interval [CI] <0), none (when 95% CI contained 0), and positive (when lower limit of 95% CI >0) together with the forest plots and presented them and their CIs in Table S2.

## Results

**Fig. 1:**
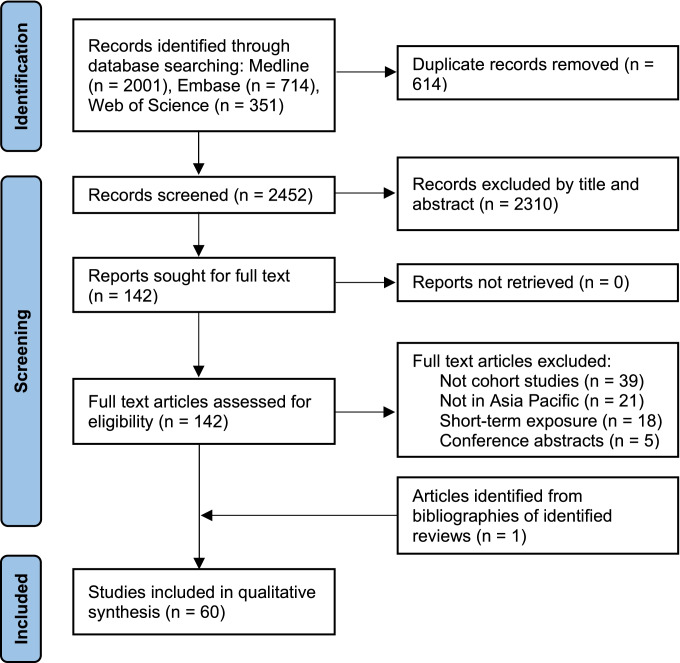
PRISMA flow diagram of identifying eligible studies of health impacts of long-term exposure to ambient PM_2.5_ in Asia Pacific, 2000–2020

After screening the titles and abstracts of 2452 records, we sought for and assessed the full text of 142 potentially eligible articles. Among these articles and the bibliographies of identified reviews, 60 studies were eligible for this systematic review (Table 1) [23–80, 81••, 82]. The PRISMA flow diagram and reasons for exclusion were presented in Fig. 1. According to the quality assessment, the included studies were all in medium (NOS score: 5–7, 24 studies) or high quality (NOS scores: 8–10, 36 studies) (Table 1 and Table S1).

**Table 1:** Characteristics of cohort studies for health effects of long-term exposure to ambient PM_2.5_ in Asia-Pacific, 2000–2020

Authors	Region	Study population	Sample size	Male, %	Age, years	PM_2.5_ assessment	PM_2.5_ exposure, μg/m^3^	Outcome	NOS score
Hanigan et al., 2019[34]	Australia	General population from DHHS database	75,268	47.6	45-54 (36.2%); 55-64 (36.5%); 65-79 (27.2%)	CTM	4.5 ± 0.6	Mortality (all-cause)	8
Hendryx et al., 2019[35]	Australia	Women from Australian Longitudinal Study on Women's Health	COPD/asthma: 31,362 /29,064	0	44.4 ± 21.0 for COPD; 47.3 ± 20.5 for asthma	Interpolated monitoring station data	Not applicable	COPD or asthma	7
Salimi et al., 2018[71]	Australia	General population from 45 and Up Study	84,285	47.8	45-54 (32.1%); 55-64 (32.2%); 65-84 (32.2%)	CTM	Mean: 4.5	Respiratory diseases	7
Chen et al., 2019[26]	China mainland	Ischemic stroke patients from China National Stroke Registry	12,291	62.0	65.5 ± 12.3	Satellite, 10 km	Mean: 80.0	Mortality (Ischemic stroke)	9
Huang et al., 2019a[37]	China mainland	General population from China-PAR project	117,575	41.0	50.9 ± 11.8	Satellite, 1 km	64.9 ± 14.2	Stroke	9
Huang et al., 2019b[38]	China mainland	General population from China-PAR project	59,456	39.0	48.4 ± 11.3	Satellite, 10 km	77.7 ± 13.2	Hypertension	9
Li et al., 2018[55]	China mainland	Elders from Chinese Longitudinal Healthy Longevity Study	13,344	42.0	89.0 (15.0)	Satellite, 1 km	Median: 50.7; range: 6.7-113.3	Mortality (all-cause)	9
Li et al., 2020a[53]	China mainland	General population from China-PAR project	118,551	41.1	51.0 ± 11.9	Satellite, 1 km	Mean: 65.0; range: 31.2-97.0	Lung cancer, mortality (lung cancer)	9
Li et al., 2020b[54]	China mainland	General population from China-PAR project	118,229	41.1	51.0 ± 11.9	Satellite, 1 km	65.0 ± 14.2	Coronary heart disease	9
Liang et al., 2019[57]	China mainland	General population from China-PAR project	88,397	39.8	51.7 ± 11.7	Satellite, 10 km	79.1 ± 13.8	Diabetes	8
Liang et al., 2020[56]	China mainland	General population from China-PAR project	116,972	41.0	51.2 ± 11.7	Satellite, 10 km	59.4 (32.6)	CVD	9
Lv et al., 2020[62]	China mainland	Elders from Chinese Longitudinal Healthy Longevity Study	15,453	43.9	92.3 ± 7.3	Satellite, 1 km	50.2 ± 13.4	Disability in activities of daily life	8
Norbäck et al., 2019[64]	China mainland	Children recruited from communities	17,679	51.0	2.0 ± 0	Interpolated monitoring station data	60.0 (9.0)	Wheeze and rhinitis	6
Peng et al., 2017[66]	China mainland	Tuberculosis patients from a mandatory reporting system	4444	74.0	<40 (26.8%); 40-60 (40.9%); >59 (32.2%)	Satellite, 10 km	53.5 (2.1)	Mortality (respiratory, respiratory cancer and diabetes)	9
Wang et al., 2020[76]	China mainland	Elders from Chinese Longitudinal Healthy Longevity Study	13,324	47.5	82.4 ± 11.9	Satellite, 1 km	50.1 (19.5)	Poor cognitive function	9
Yang et al., 2020[78]	China mainland	General population from China-PAR project	116,821	41.0	51.6 ± 11.7	Satellite, 1 km	64.9 ± 14.2	Mortality (non-accidental and cardio-metabolic)	9
Yin et al., 2017[80]	China mainland	Males >40 years-old selected from 145 Disease Surveillance Points	189,793	100	54.8 ± 10.7	Satellite and CTM, 10 km	Mean: 43.0	Mortality (non-accidental, CVD, cerebrovascular, COPD, and lung cancer)	9
Qiu et al., 2017[68]	Hong Kong	Elders that visited Elderly Health Centers	61,447	34.1	72.1 ± 5.6	Satellite, 1 km	35.8 ± 2.4	Stroke	7
Qiu et al., 2018[67]	Hong Kong	Elders that visited Elderly Health Centers	53,905	34.2	72.1 ± 5.7	Satellite, 1 km	37.6 ± 2.8	Type 2 diabetes	7
Ran et al., 2020a[69]	Hong Kong	Elder CKD patients that visited Elderly Health Centers	902	42.1	72.8 ± 6.0	Satellite, 1 km	37.8 ± 2.9	Mortality (all-cause, CVD, stroke, respiratory, renal)	6
Ran et al., 2020b[70]	Hong Kong	Elders that visited Elderly Health Centers	61,447	34.1	72.0 ± 5.6	Satellite, 1 km	35.8 (3.2)	Mortality (Renal)	5
Sun et al., 2020[74]	Hong Kong	Elders that visited Elderly Health Centers	58,643	34.3	71.9 ± 5.5	Satellite, 1 km	Median: 35.3	Mortality (cardiovascular and respiratory)	7
Yang et al., 2018[79]	Hong Kong	Elders that visited Elderly Health Centers	61,386	32.6	70.2 ± 5.5	LUR model	42.2 (5.5)	Mortality (all-cause, CVD, respiratory)	7
Han et al., 2020[33]	South Korea	General population from National Health Insurance Research Database	687,940	42.5	31.2 ± 4.0	CTM	31.2 ± 4.0	COPD	9
Kim et al., 2016[47]	South Korea	General population National Health Insurance Research Database	27,270	54.0	15-39 (24.0%); 40-59 (57.0%); 60-79 (19.0%);	Monitoring station	29.9 ± 3.5	Major depressive disorder	8
Kim et al., 2017[44]	South Korea	General population from NHIS-NSC	136,094	49.1	42.1 ± 14.8	Monitoring station	Mean: 25.6, IQR: 1.5	Cardiovascular mortality and events	8
Kim et al., 2019[46]	South Korea	General population from NHIS-NSC	432,587	50.1	18-34 (22.0%); 35-49 (35.0%); 50-64 (29.0%); >64 (14.0%)	Monitoring station	Not reported	Atrial fibrillation	8
Kim et al., 2020a[45]	South Korea	General population from NHIS-NSC	436,933	50.1	Range: 18-75 Mean: 47.8	Monitoring station	Mean: 18.8	Mortality (all-cause and CVD)	8
Kim et al., 2020b[48]	South Korea	General population from NHIS-NSC	196,167	53.5	46.6 ± 11.0	Monitoring station	52.3 ± 6.2	Cardiovascular disease	8
Lee et al., 2019[51]	South Korea	General population from NHIS-NSC	119,998	55.3	55.1 ± 7.1	3D photochemical air quality model	23.6 (14.0)	Metabolic syndrome	9
Noh et al., 2019[63]	South Korea	General population from NHIS-NSC	62,676	49.3	20-39 (31.7%); 40-49 (29.1%); >49 (39.3%)	Monitoring station	Rang: 25.1-38.9	Hemorrhagic Stroke	8
Shin et al., 2020a[72]	South Korea	General population from NHIS-NSC	115,728	47.2	60.0 ± 7.2	Monitoring station	Not reported	Senile cataract	8
Shin et al., 2020b[73]	South Korea	General population from NHIS-NSC	85,869	50.8	20-39 (25.1%); 40-64 (60.4%); >64 (14.6%)	Monitoring station	25.9 ± 3.6	Fasting blood glucose and lipid profiles	8
Zhang et al., 2019[82]	South Korea	Population undergoing regular health examinations from KSCS cohort	123,045	60.1	39.4 ± 6.8	LUR model	24.3 ± 1.3	Depression	6
Zhang et al., 2020 [81••]	South Korea	Population undergoing regular health examinations from KSCS cohort	182,488	56.3	36.5 ± 7.0	LUR model	26.6 ± 2.3	Cardiac arrhythmia	7
Bo et al., 2019[23]	Taiwan	General population from Taiwan MJ cohort	66,702	45.4	38.5 ± 12.1	Satellite, 1 km	27.1 ± 8.1	Dyslipidemia	7
Chan et al., 2018[24]	Taiwan	General population years from Taiwan MJ cohort	100,629	52.5	38.9 ± 11.3	Satellite, 1 km	27.1 ± 8.0	Chronic kidney Disease	7
Chang et al., 2016[25]	Taiwan	General population National Health Insurance Research Database	244,413	45.6	31.0 ± 18.0	Monitoring station	Mean: 33.3	Rheumatoid arthritis	8
Chen et al., 2020[27]	Taiwan	Elders from a senior health checkup program	360	46;0	71.9 ± 4.9	Interpolated monitoring station data	Mean: 29.1	Cognitive impairment	6
Chin et al., 2018[28]	Taiwan	Type 2 diabetes patients from 36 local clinics	812	46.1	55.4 ± 8.4	Interpolated monitoring station data	34.1 ± 6.0	Microalbuminuria,	8
Fan et al., 2018[29]	Taiwan	General population from National Health Insurance Research Database	162,797	43.9	40.5 ± 14.6	Monitoring station	34.9 ± 8.8	Nasopharyngeal carcinoma	8
Guo et al., 2018[32]	Taiwan	General population from Taiwan MJ cohort	91,709	49.8	41.6 ± 13.1	Satellite, 1 km	26.7 ± 7.8	Lung function and COPD	7
Guo et al., 2020a[30]	Taiwan	General population from Taiwan MJ cohort	385,650	48.6	39.6 ± 13.0	Satellite, 1 km	26.6 ± 7.6	Mortality (gastrointestinal cancer)	7
Guo et al., 2020b[31]	Taiwan	General population from Taiwan MJ cohort	140,072	48.6	39.5 ± 10.7	Satellite, 1 km	26.6 ± 7.6	Hypertension	7
Hong et al., 2020[36]	Taiwan	Children from National Health Insurance Research Database	218,008	52.0	6.0 ± 3.0	Monitoring station	Mean: 34.7	Recurrent headache	7
Huang et al., 2014[39]	Taiwan	Patients undergoing peritoneal dialysis	175	28.6	49.8 ± 10.8	Monitoring station	29.6 (3.4)	Dialysis-related infection	6
Hwang et al., 2015[40]	Taiwan	Children from 14 communities	2941	52.1	12.0 ± 0	Interpolated monitoring station data	34.5 ± 9.1	Lung function	7
Jung et al., 2015[43]	Taiwan	Elders from National Health Insurance Research Database	95,690	53.9	74.0 (9.0)	Interpolated monitoring station data	33.6 ± 9.2	Alzheimer’s Disease	8
Jung et al., 2019a[41]	Taiwan	Infants from Taiwan Maternal and Child Health Database	184,604	59.0	0 ± 0	Satellite, 10 km	35.6 ± 3.5	Asthma	9
Jung et al., 2019b[42]	Taiwan	General population National Health Insurance Research Database	682,208	50.9	38.0 (19.0)	Satellite, 1 km	34.4 ± 7.6	Systemic lupus erythematosus	9
Lai et al., 2016[49]	Taiwan	Participants of a voluntary community-based integrated screening program	106,678	35.1	50.8 (16.6)	Monitoring station	27.5 ± 3.4	Tuberculosis	6
Lao et al., 2019[50]	Taiwan	General population from Taiwan MJ cohort	147,908	50.1	38.3 ± 11.5	Satellite, 1 km	26.8 ± 7.8	Type 2 diabetes	7
Li et al., 2019[52]	Taiwan	General population National Health Insurance Research Database	505,151	48.7	42.6 ± 15.8	LUR model	Mean: 27.9	Type 2 diabetes	9
Lin et al., 2018[58]	Taiwan	General population National Health Insurance Research Database	161,970	43.8	40.5 ± 14.6	Monitoring station	34.8 ± 8.76	Nephrotic Syndrome	8
Lin et al., 2019[60]	Taiwan	General women from National Health Insurance Research Database	91,803	0	36.9 ± 18.8	Interpolated monitoring station data	30.9 ± 6.2	Polycystic Ovary Syndrome	8
Lin et al., 2020a[61]	Taiwan	General population National Health Insurance Research Database	161,970	43.8	37.9 (20.3)	Interpolated monitoring station data	33.3 (11.7)	Chronic kidney Disease	9
Lin et al., 2020b[53]	Taiwan	CKD patients from National Advanced CKD registry	6628	57.6	67.8 (19.1)	Satellite, 3 km	36.3 (7.8)	Renal failure with replacement therapy	9
Pan et al., 2015[65]	Taiwan	General population recruited from 7 townships	22,062	50.4	30-39 (34.3%); 40-49 (26.6%); 50-65 (39.0%)	Interpolated monitoring station data	Medians in two sites: 36.0/24.1	Hepatocellular carcinoma	7
Tseng et al., 2015[75]	Taiwan	Civil service employees and teachers from Civil Servants cohort	42,599	57.0	41.3 ± 10.5	Monitoring station	P_20_-P_80_: 27.3-30.9,	Mortality (all-cause, CVD, and cerebrovascular)	6
Wei et al., 2019[77]	Taiwan	Children from National Health Insurance Research Database	97,306	52.7	8.7 ± 1.7	Monitoring station	33.6 (11.7)	Myopia	8

Age and PM_2.5_ exposure are given as mean ± SD or median (IQR) or range (proportion) or as described

*PM*_*2.5*_ particulate matter with an aerodynamic diameter ≤2.5 μm, *CHD* coronary heart disease, *CKD* chronic kidney diseases, *CVD* cardiovascular diseases, *COPD* chronic obstructive pulmonary disease, *IDW* inverse distance weighting, *CTM* chemical transport model, *LUR* land use regression, *SD* standardized deviation, *IQR* interquartile range

**Fig. 2:**
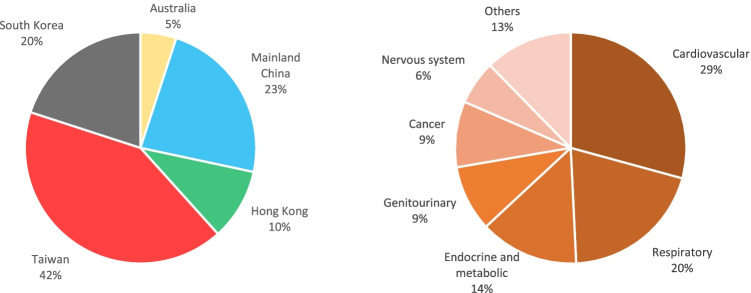
Distribution of countries/regions (left) and outcomes (right) among included studies

Of the included studies, 50% were based on administrative datasets (e.g., national insurance dataset); only 12% ascertained outcomes totally or partially through questionnaires, 47% ascertained outcomes totally or partially through register-based data (of which 43% were studies about morbidity, and 57% were about mortality), 33% ascertained outcomes based on examination results; exposures were derived by linking addresses of residence/school/hospital to satellite-based models (58%), models (e.g., chemical transport model) based on monitoring stations network (13%), or data recorded by air pollution monitoring stations (28%). These studies were conducted in five countries/regions (Fig. 2).

Individual mean exposure to ambient PM_2.5_ was the lowest in Australia (around 4.5 μg/m^3^) and highest in Mainland China (> 50 μg/m^3^) as presented in Table 1. Most of the study populations were from the general population that consisted of both sexes, except six from patients [26, 28, 39, 61, 66, 69], one from infants [41], four from children [36, 40, 64, 77], six from elderly people [43, 67, 68, 70, 74, 79], and three studies just including males or females [35, 60, 80]. Lifestyle (i.e., cigarette or alcohol consumption) was considered as covariates in 81.7% of the reviewed studies, and individual-level socioeconomic status (i.e., education or income) was adjusted in 87% of the reviewed studies. Only five studies adjusted for environmental factors other than air pollution (e.g., temperature and greenspace) [38, 46, 51, 57, 64].

**Fig. 3:**
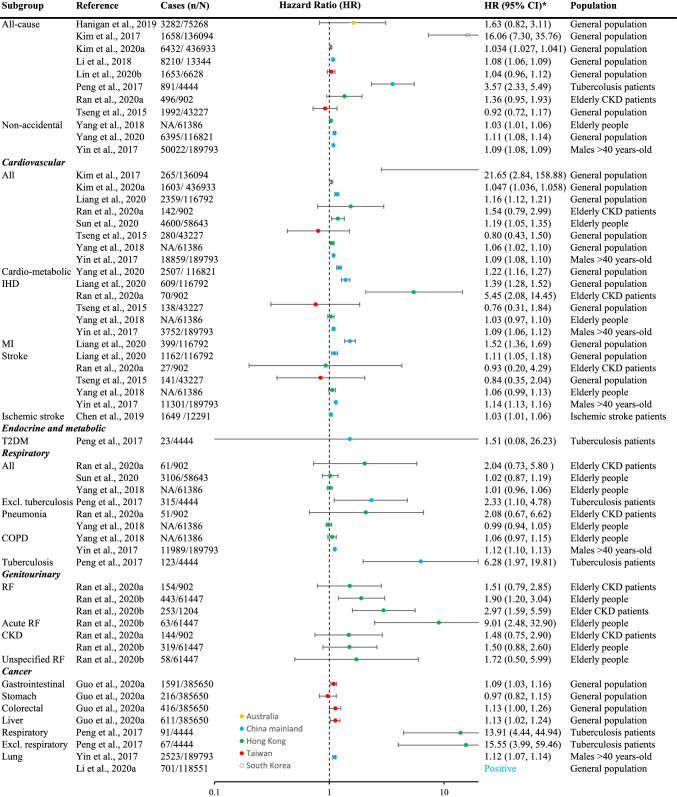
Mortalities associated with each 10 μg/m^3^ increase in long-term exposure to ambient PM_2.5_ in Asia Pacific cohorts studies, 2000-2020. IHD = Ischemic heart diseases, MI = myocardial infarction, T2DM = type 2 diabetes mellitus, COPD = chronic obstructive pulmonary disease, RF = renal failure, CKD = chronic kidney diseases. * Negative (upper limit of 95% confidence interval [CI] <0), none (95% CI contains 0), and positive (lower limit of 95% CI >0); and the font color indicates the region, which is in line with the legend of forest plots

**Fig. 4:**
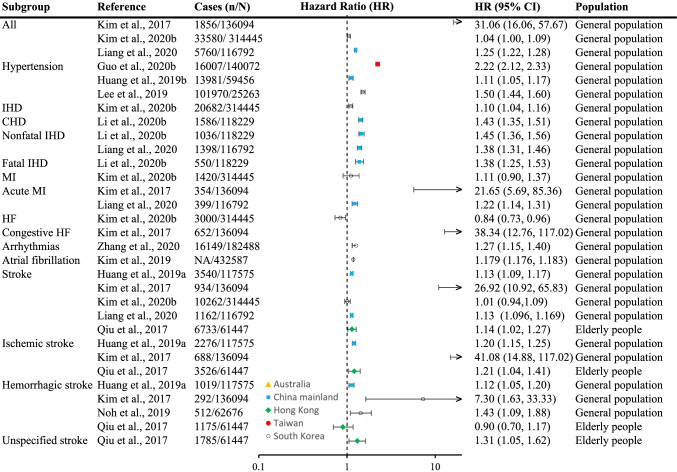
Cardiovascular disease incidences associated with each 10 μg/m^3^ increase in exposure to long-term exposure to ambient PM_2.5_ in Asia Pacific cohorts studies, 2000-2020. IHD = ischemic heart diseases; CHD = coronary heart diseases; MI = myocardial infarction, HF = heart failure

**Fig. 5:**
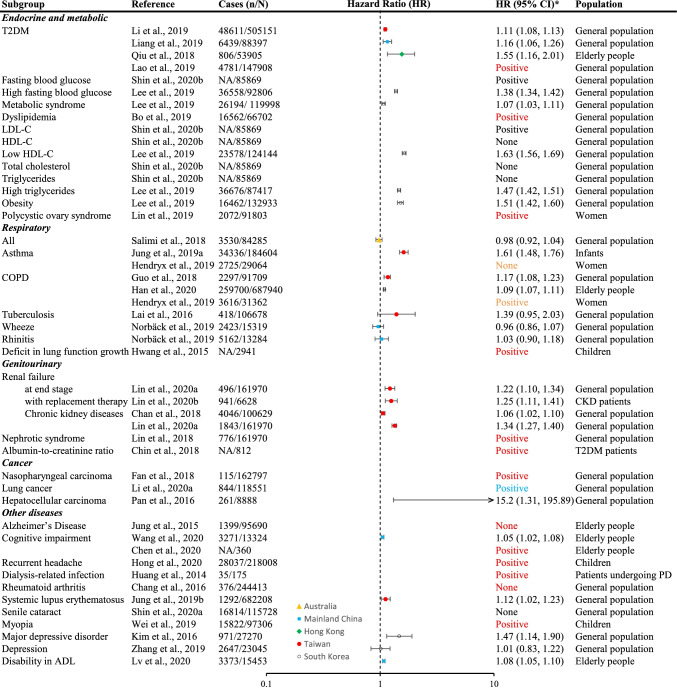
Disease incidences associated with each 10 μg/m^3^ increase in long-term exposure to ambient PM_2.5_ in Asia Pacific cohorts studies, 2000-2020. T2DM = type 2 diabetes mellitus, LDL-C = low-density lipoprotein cholesterol, HDL-C = high-density lipoprotein cholesterol, COPD = chronic obstructive pulmonary disease, ADL = activities of daily living, CKD = chronic kidney diseases, PD = peritoneal dialysis. *Negative (upper limit of 95% confidence interval [CI] <0), none (95% CI contains 0), and positive (lower limit of 95% CI >0); and the font color indicates the region, which is in line with the legend of forest plots. Norbäck et al. (2019) and Chen et al. (2020) reported odds ratios

The included studies assessed the effects of long-term exposure to ambient PM_2.5_ on the incidence of various diseases, as well as all-cause/nonaccidental and cause-specific mortalities (Table 1). The distribution of these outcomes was demonstrated in Fig. 2. All estimates plotted in forest plots (Figs. 3, 4, 5) were yielded from single-polltant models. Although there were two studies only presented multi-pollutant models, the exposures of these models were categorical. Therefore, their effect estimates were not plotted in the forest plots [28, 39]. Overall, the effect estimates of 17 studies were not plotted in forest plots but were summarized in Table S2.

We identified a study possibly with crucial flaws. All effect estimates (HRs ranged in 7.30–41.08) reported by Kim et al. [44] were implausibly high compared with the effect estimates from other studies. This study was based on 136,094 insurants randomly selected in Seoul, South Korea. Individual exposure (mean: 25.6 μg/m^3^, IQR: 1.5 μg/m^3^) to ambient PM_2.5_ was defined as the mean PM_2.5_ concentration of the monitoring stations with the same postcode as the individual’s residential address. Outcomes were ascertained through death registration or hospitalization records. BMI and lifestyle were not adjusted in statistical models.

### Mortality

The effect of long-term exposure to ambient PM_2.5_ on mortality was investigated by 17 cohort studies (Fig. 3). The effect on all-cause/nonaccidental mortality (*n*=11) was assessed in Australia (one study) [34], Mainland China (four studies) [55, 66, 78, 80], Hong Kong (two studies) [61, 75], Taiwan (two studies) [69, 79], and South Korea (two studies) [44, 45]. Seven of these studies found that long-term exposure to ambient PM_2.5_ increased all-cause/non-accidental mortality in the general population [44, 45, 78, 80], in elderly people [79], in chronic kidney diseases patients [69], and in tuberculosis patients [66]. The magnitudes of the effects reported by the seven studies were similar (HR varied from 1.03 to 1.11) except for the effects reported by the possibly flawed study in South Korea [44] and a study in Mainland China [66]. The study in Mainland China [66] found a strong effect on all-cause mortality (HR: 3.57, 95% CI: 2.33–5.49) in a cohort of tuberculosis patients recruited from four districts in Shanghai (*N* = 4,444). Individual exposure (median: 53.5 μg/m^3^, IQR: 2.1 μg/m^3^) was assessed based on satellite data with a spatial resolution of 10 km × 10 km. Body mass index (BMI), lifestyle (except smoking), and individual-level socioeconomic status (SES) were not adjusted in statistical analyses.

The effect of long-term exposure to ambient PM_2.5_ on CVD mortality was assessed in Mainland China (four studies) [26, 56, 78, 80], Hong Kong (three studies) [69, 74, 79], Taiwan (one study) [75], and South Korea (two studies) [44, 45], and only one study [75] did not observe any association (Fig. 3). An effect estimate on ischemic heart diseases mortality (HR: 5.45, 95% CI: 2.08–14.45) was huge compared with the estimates reported by other studies. It was observed among 902 elderly chronic kidney diseases patients with previous hospitalization history in Hong Kong [69]. Individual exposure (mean: 37.8, SD: 2.9) was assessed based on satellite data with a spatial resolution of 1 km × 1 km.

The effect of long-term exposure to ambient PM_2.5_ on respiratory mortality was only investigated in Mainland China (two studies) [66, 80] and Hong Kong (three studies) [69, 74, 79]. No associations were found among elderly people [69, 74, 79]. Associations were only observed by two studies. The study conducted among 4,444 tuberculosis patients from four districts in Shanghai, China [66] found the mortalities of tuberculosis and other respiratory diseases increased by 6.28 (95% CI: 1.97–19.81) and 2.33 (95% CI: 1.10–4.78) times per 10 μg/m^3^ increase in long-term exposure to ambient PM_2.5_ (median: 53.5 μg/m^3^, IQR: 2.1 μg/m^3^), and Yin et al. [80] found the mortality of chronic obstructive pulmonary disease increased by 12% (95% CI: 10–13%) per 10 μg/m^3^ increase in long-term exposure to ambient PM_2.5_ among randomly selected males who were >40 years-old. Both studies assessed individual exposure by employing satellite data with a spatial resolution of 10 km × 10 km.

The effect of long-term exposure to ambient PM_2.5_ on cancer mortality was assessed in Mainland China (two studies) [53, 80], Hong Kong (one study) [66], and Taiwan (one study) [30]. Although every study had observed an association between ambient PM_2.5_ exposure and cancer mortality, the evidence was limited for each kind of cancer in terms of the number of studies (Fig. 3).

Only the effect on kidney diseases was studied among all genitourinary diseases, and it was only investigated by Ran et al. [69, 70] based on one cohort consisting of elderly people in Hong Kong (Fig. 3). They found that the renal failure mortality was increased by long-term exposure to ambient PM_2.5_, and the effect was stronger among those with chronic kidney disease (CKD) at baseline. Notably, they observed that the mortality from acute kidney injury increased by nine times for each 10 μg/m^3^ increase in long-term exposure to ambient PM_2.5_ in general elderly people. Lifestyle, individual- and district-level SES, and pre-existing diseases were adjusted in statistical analyses.

As shown in Fig. 3, the effect of long-term exposure to ambient PM_2.5_ on the mortality from type 2 diabetes mellitus (T2DM) was only assessed by the study that was based on 4,444 tuberculosis patients from four districts in Shanghai [66]. The result indicated that long-term exposure to ambient PM_2.5_ was not associated with T2DM mortality (HR: 1.51, 95% CI: 0.08–26.23).

### Cardiovascular diseases

The effect of long-term exposure to ambient PM_2.5_ on the incidence of CVD was estimated by 12 cohort studies in Mainland China (four studies) [37, 38, 54, 56], Hong Kong (one study) [68], Taiwan (one study) [31], and South Korea (six studies) [44, 46, 48, 51, 63, 81••] (Fig. 4). Overall, every study had found an association, indicating the incidence of CVD increased with long-term exposure to ambient PM_2.5_. All effect estimates were comparable, except for the effects reported by the possibly flawed study [44].

As for specific CVD, the associations observed for the incidences of hypertension [31, 38, 51] and ischemic heart diseases [44, 48, 54, 56] were consistent. Stroke was the most studied CVD, and its risk consistently increased with higher exposure except that only one study did not observe any association [48]. The investigation of the effects on other CVD, including heart failure and arrhythmias, was relatively poor in amount.

### Endocrine and metabolic diseases

Seven cohort studies investigated the effects of long-term exposure to ambient PM_2.5_ on the incidence of endocrine and metabolic diseases, and one had assessed the effects on the levels of fasting glucose and lipid profiles (Fig. 5). All of them had found evidence indicating that long-term exposure to ambient PM_2.5_ increased the risk of endocrine and metabolic diseases. T2DM was investigated in Mainland China (one study) [57], Hong Kong (one study) [67], and Taiwan (two studies) [50, 52], and all of these studies found that the incidence of T2DM increased with higher exposure. While two studies ascertained incidence of T2DM using insurance or hospitalization records and might have missed undiagnosed cases [52, 67], T2DM was diagnosed through blood tests for every participant in the other two studies [50, 57].

Although other diseases, including metabolic syndrome [51], dyslipidemia [23], obesity [51], high fasting blood glucose [51], and polycystic ovary syndrome [60], were relatively less investigated in terms of the amount of studies, the findings consistently supported the association between long-term exposure to ambient PM_2.5_ and metabolism. In addition, Shin et al. [73] found that fasting blood glucose and low-density lipoprotein cholesterol increased following long-term exposure to higher ambient PM_2.5_.

### Respiratory diseases

We identified eight cohort studies that had assessed the effects of long-term exposure to ambient PM_2.5_ on respiratory diseases morbidities (Fig. 5). The association with chronic obstructive pulmonary disease (COPD) was observed in general population, elderly people, and women [32, 33, 35]. The association with asthma was assessed in infants and women by two studies respectively but was only found in infants [35, 41]. Although the association with deficit in lung function growth in children was found by a study, the sample size was small (*N* = 2941) [40]. The effects on other outcomes, including the morbidities of all respiratory diseases, tuberculosis, wheeze, and rhinitis were only assessed by one study individually.

### Genitourinary diseases

Only the effect on kidney diseases morbidities were studied among all genitourinary diseases (Fig. 5). Although relevant studies were only conducted in Taiwan, the findings consistently supported that long-term exposure to ambient PM_2.5_ could increase the incidence of kidney diseases. Two studies [24, 59] assessed the effect on chronic kidney diseases (CKD) among general population. Both found that the incidence of CKD increased because of long-term exposure to ambient PM_2.5_. In addition, the effect still existed for the incidence of end-stage renal failure among general population [59] and the incidence of renal failure with replacement therapy among CKD patients [61]. In another study, Lin et al. [58] observed that long-term exposure to ambient PM_2.5_ increased the risk of nephrotic syndrome based on a national insurance dataset, although the individual exposure was only assessed through monitoring station data. In concert with above findings, Chin et al. [28] observed that albumin-to-creatinine ratio was elevated by long-term exposure to ambient PM_2.5_ among 812 T2DM patients recruited from 36 clinics in Northern, Central, and Southern Taiwan.

### Other health effects

The investigation about the effects on other diseases morbidities were limited. Associations were found for diseases including cancers [29, 53, 65], cognitive impairment [27, 76], recurrent headache [36], dialysis-related infection [39], systemic lupus erythematosus [42], myopia [77], major depressive disorder [47], and disability in activities of daily living [62]. On the other hand, the associations with Alzheimer’s Disease [43], rheumatoid arthritis [25], senile cataract [72], and depression [82] were not found.

## Discussion

Through a systematic review of cohort studies covering a broad range of health effects of long-term exposure to ambient PM_2.5_ in Asia-Pacific, we identified 60 eligible studies. These studies investigated the incidences of cardiovascular diseases (CVD), endocrine and metabolic diseases, respiratory diseases, genitourinary diseases, cancers, nervous system disorders, infectious diseases, autoimmune diseases, eye diseases, mental disorders, disability in activities of daily living, as well as all-cause/nonaccidental and cause-specific (cardiovascular, endocrine, respiratory, genitourinary, and cancer) mortalities. Through synthesizing these studies, we found consistent evidence supporting that long-term exposure to ambient PM_2.5_ increased all-cause/non-accidental and CVD mortality as well as the incidences of CVD, T2DM, kidney diseases, and COPD. We also identified inequalities in PM_2.5_-health associations and some research gaps.

### Current evidence

The biological plausibility of the health effects of PM_2.5_ has been well established by previous studies [83]. In general, inhaled PM_2.5_ and its components lead to injury, inflammation, and oxidative stress through interacting with the cells in respiratory tract. North et al. [19•] had reviewed the sources, health burden, respiratory effect of air pollution in Asia-Pacific. This work was a quick guide with a broad scope but not able to provide comprehensive evidence and deep insights because (1) it was a general review and did not perform systematic literature searches; (2) it just covered the respiratory effect of air pollution, while air pollution had been associated with a various range of health outcomes; (3) it did not differentiate the health effects of different air pollutants, while they could vary significantly. According to this systematic review, we presented an extensive range of health effects of long-term exposure to ambient PM_2.5_ from cohort studies in Asia-Pacific, which was comprehensive evidence for local governments in this region. This has not been done previously and is beneficial for future cost-effectiveness analyses, which is crucial for policymaking. In addition, since we had identified all cohort studies about the health effects of long-term exposure to ambient PM_2.5_ in Asia-Pacific, research gaps were able to be found and discussed in the following sections.

#### All-cause and cause-specific mortalities

Through this systematic review, we conclude that the evidence clearly demonstrates that all-cause/non-accidental and CVD mortalities increased with long-term ambient PM_2.5_ exposure; long-term ambient PM_2.5_ exposure was generally associated with higher respiratory mortality; the associations of long-term ambient PM_2.5_ exposure with the mortalities of renal diseases and cancer were suggested, though further research was warranted.

According to our literature search, the latest systematic review about health effects of long-term exposure to ambient PM_2.5_ in Asia-Pacific before our work summarized all epidemiological evidence in China (including Hongkong and Taiwan) that were published before 2013 [13]. That review found no cohort studies had been conducted previously. Our findings demonstrated that the evidence base from cohort studies had rapidly increased during 2014-2020. The findings reported by the cohort studies in Asia-Pacific were generally consistent with studies conducted in other regions. Chen and Hoek [20] found the evidence published before 2018 supported that long-term exposure to ambient PM_2.5_ increased non-accidental (pooled RR: 1.08, 95% CI: 1.06–1.09), CVD (pooled RR: 1.11, 95% CI: 1.09–1.14), and respiratory (pooled RR: 1.10, 95% CI: 1.03, 1.18) mortalities through a meta-analysis, which included 104 cohort studies and three case-control studies conducted in Europe, America, and Western Pacific. Bowe et al. [9] observed the association between ambient PM_2.5_ exposure and increased mortalities of chronic kidney disease in the USA. According to the latest Integrated Science Assessment [83], previous studies provided consistent evidence supporting the association of long-term exposure to ambient PM_2.5_ with lung cancer mortality, whereas the studies about the associations for the mortalities from other cancers were scarce.

#### Disease incidences

Cohort studies in Asia-Pacific demonstrated that long-term ambient PM_2.5_ exposure increased the risks of CVD, T2DM, kidney diseases, and COPD. The effect estimates for CVD morbidity were consistent. According to the forest plots, the health effect on CVD morbidity was slightly higher than the effect on CVD mortality. The findings in Asia-Pacific were generally consistent with the findings in Europe. A pooled analysis of cohorts from the European Study of Cohorts of Air Pollution Effects (ESCAPE) project demonstrated that the incidence of coronary heart diseases (CHD) was increased by long-term exposure to ambient PM_2.5_ [84•], while another pooled analysis from the Effects of Low-level Air Pollution (ELAPSE) project, which was built on the ESCAPE project, observed the association of the incidence of stroke, although the association of CHD missed [85]. The effect estimates reported by the reviewed studies were comparable to pooled effects synthesized by previous meta-analyses. The pooled HR for stroke incidence was 1.23 (95% CI: 1.11–1.37) in North America and Europe [2•]; the pooled HR of myocardial infarction synthesizing cohort studies before 2020 was 1.10 (95% CI: 1.02–1.18) [1•]; the pooled HR of T2DM was 1.11 (95% CI: 1.03, 1.19) in American countries [86]; and the pooled HR of COPD was 1.18 (95% CI: 1.13–1.23) [4]. Cohort studies in the USA consistently supported the associations of long-term ambient PM_2.5_ exposure with a declined renal function as well as increased incidences and progression of kidney diseases [87, 88].

Although research of the associations of long-term ambient PM_2.5_ exposure with other diseases was limited in terms of the number of studies in Asia-Pacific, it was generally consistent with the studies conducted elsewhere. According to a meta-analysis, long-term exposure to ambient PM_2.5_ was associated with increased risk of lung cancer, while the knowledge gaps of the effects on other cancers still existed [8••]. As for respiratory diseases other than COPD, the Children’s Health Study conducted in the USA had provided convincing evidence of the association between long-term exposure to ambient PM_2.5_ and decrement in lung growth of children [89, 90], and some studies had observed the associations of long-term ambient PM_2.5_ exposure with lung function decline and asthma, especially in children [91–95]. These findings are accordant to the association of asthma among infants observed by Jung et al. [41].

#### Exceptional estimates

We identified three studies that reported much stronger effect estimates compared to other studies [44, 66, 69]. All effects estimated by the study conducted by Kim et al. in Seoul [44] were implausibly higher than the effects found by other studies. This might be the result of the imprecise exposure assessment. In this study, the individual exposure was defined as the records of the air quality monitoring station in the same district as the individual’s residence, and the population density was high in Seoul, which could cause serious exposure misclassification. Given the individual PM_2.5_ exposure varied within a narrow range (IQR: 1.5 μg/m^3^_,_ range: 23.8–27.8 μg/m^3^), the observed effects might be significantly biased by the exposure misclassification and then be inflated by extrapolating to an exposure scale (i.e., 10 μg/m) that was much larger than the observed exposure range. The study conducted by Peng et al. [66] in Shanghai provided relatively high estimates of all-cause mortality and some cause-specific mortalities. One reason might be the participants were tuberculosis patients, which might had already been more vulnerable to PM_2.5_. Therefore, the effect estimates would be higher than the effects observed in general population. Meanwhile, the effect estimates might have been biased by not adjusting for some covariates (i.e., BMI, lifestyle, and SES), as well as the exposure assessment method. The participants were sourced from merely four districts of this city, and the areas of two of the four district, Putuo and Yangpu were only 55 and 60 km^2^. In this occasion, the spatial resolution of the satellite data (10 km × 10 km) used for exposure assessment was too low to distinguish individual exposure, which would lead to a low individual exposure variability (IQR: 2.1 μg/m^3^ = 4% of median). The other study was performed in Hong Kong by Ran et al. [69] and reported a relatively higher effect estimate for ischemic heart disease mortality. The reasons might be that (1) the participants were elderly CKD patients with hospitalization history, which might have been more vulnerable to the exposure; (2) the individual exposure variability was moderate (SD = 8% of mean); (3) sample size (*N* = 902) was too small to provide a sufficient statistical power.

### Inequalities in PM_2.5_-health associations

#### Inequalities in vulnerability

PM_2.5_-related health effects can vary across populations [96], since some populations may be more vulnerable to PM_2.5_. According to the stratified analyses of some reviewed studies, the adverse health effects of long-term exposure to ambient PM_2.5_ on mortality were constantly stronger in smokers [30, 45, 80], obese and overweight people [26, 79], and people with pre-existing CVD [45, 69, 78]. The elderly [46, 54, 56, 63, 68, 81••] and people with pre-existing CVD [46, 48, 54] or obesity [46, 48, 63] were also consistently more vulnerable to the cardiovascular effect of long-term exposure to ambient PM_2.5_. As summarized by the latest Integrated Science Assessment (ISA) for Particulate Matter [83], similar findings have been reported in the USA. However, while ethnic minorities and children were found more vulnerable to PM_2.5_-related health effects in the USA [83], few cohort studies have assessed the vulnerability of ethnic minorities and children in Asia-Pacific. In addition, some cohort studies in Asia-Pacific reported that the health effects of long-term ambient PM_2.5_ exposure varied by PM_2.5_ exposure level [28, 55], alcohol consumption [50, 55, 65], physical activity [31, 72, 81••], education level [27], sex [27, 32, 36, 40, 46, 48, 51, 57, 62, 65, 67, 70, 79], and region of residence (i.e., urban or rural area) [48, 55–57, 80], but the findings were inconsistent or insufficient to make an inference.

#### Inequities in exposure

PM_2.5_ exposure level could be unequally distributed across subpopulations in Asia-Pacific, even if occupational exposure is not considered. According to the studies included in this review, ambient PM_2.5_ concentration was lower in rural areas compared to that in urban areas in China [55, 80]. The pattern in the USA and Europe was the same [97•, 98]. This might be the result of expanding traffic, industry, and energy production in urban and suburb areas and implied that urban residents had more health burden from ambient PM_2.5_ than rural residents. However, rural residents might be exposed to more indoor PM_2.5_ than urban residents due to combustion of polluting fuels (e.g., wood, coal, and kerosene) for purposes of cooking and heating, where the indoor PM_2.5_ can reach >60 time higher than WHO guideline [99]. According to the PURE-AIR study, 57% and 43% of the households were still using polluting fuels as primary fuel in India and China [99], which were the most populous countries in Asia-Pacific, owning 36% of the world population. Therefore, in Asia-Pacific rural areas, more attention should be given to indoor PM_2.5_ exposure when implementing air pollution mitigation and adaptation policies. Moreover, females may be exposed to a higher PM_2.5_ level because they spend more time in kitchen than males [99]. Previous evidence also suggested that a lower household income was associated with a higher residential air pollution level in urban areas [100]. However, more evidence is requested to prove these inequities in PM_2.5_ exposure.

### Research gaps

As the top 20 causes of death, diabetes mellitus, kidney diseases, dementia, and lung cancers led to 9% and 13.7% of death in South-East Asia and Western Pacific in 2019 [101]. Long-term exposure to ambient PM_2.5_ was associated with these diseases, according to studies conducted outside of Asia-Pacific. [5, 6, 8••, 9, 102]. However, only scarce cohort studies had been conducted to investigate the associations of long-term exposure to ambient PM_2.5_ with the mortalities of these diseases in Asia-Pacific according to our systematic review.

On the other hand, the investigations of some associations were only performed in selective countries or populations. For example, the association of long-term exposure to ambient PM_2.5_ with respiratory mortality was only investigated in Mainland China and Hong Kong, and the study populations were restricted to elderly people or those with pre-existing diseases; and the association with kidney diseases morbidity was only investigated in Taiwan. Research of these associations should be expanded to understudied countries in Asia-Pacific.

Previous studies indicated that the health effect of long-term exposure to ambient PM_2.5_ varied with concentration of PM_2.5_ and populations [55, 74]. Therefore, it is crucial to have relevant studies distributed across regions with various levels of PM_2.5_ and populations to reveal the whole spectrum of the PM_2.5_-related health effects. However, almost all studies included in our systematic review were conducted in regions with a moderate-to-high PM_2.5_ concentration (>25 μg/m^3^). Given the most recent studies indicated that there was no threshold for the health effects of long-term exposure to ambient PM_2.5_ [3, 10, 103••, 104], relevant studies should also be conducted in countries with low ambient PM_2.5_, including Australia, New Zealand, Japan, Malaysia, and Philippines, as well as countries with moderate ambient PM_2.5_, such as Singapore, Indonesia, and selective provinces of China (e.g., Sichuan, Yunnan, and Hainan). Future cohort studies should also be conducted in India and Pakistan, which are both populous countries with high levels of air pollution.

In addition, some issues emerged from the reviewed cohort studies and should be noted when studies are designed in the future. First, half of these studies were based on administrative datasets (e.g., national insurance datasets). These studies had strengths in terms of sample sizes and expenses but also had limitations because participants’ lifestyle information and SES were usually unavailable. However, failing to adjust them may introduce biases when estimating the health effects of long-term exposure to ambient PM_2.5_ [105, 106]. Second, environmental factors have been solidly associated with both human health and PM_2.5_¸which may confound the associations of interest [107–109]. However, 93% of the reviewed studies did not consider environmental factors (e.g., temperature, humidity, and greenspace) as covariates [46, 51]. Third, 12 studies ascertained new cases of diseases based on register-based datasets (e.g., inpatient/outpatient dataset) and might omit some cases. Biases would be introduced when new cases with mild symptoms were not captured because hospitalizations were not necessary while the severity of symptoms was associated with the exposure level, or participants’ low SES influenced their willingness or access to health services, considering SES was associated with ambient PM_2.5_ exposure [105]. Fourth, about 28% of the reviewed studies assessed individual exposure merely using air pollution data collected from monitoring stations. Even though some of them calculated residential exposure through inversed distance weighting, significant exposure misclassifications were still possible because the diffusion of air pollutants is influenced by various factors including land use, wind speed and direction, temperature, and topography [110]. Therefore, advanced models that incorporate comprehensive sets of factors are warranted for precise individual exposure assessment and hence will essentially improve estimations of PM_2.5_-related health effects, investigations on population vulnerability, and analyses of exposure inequities.

Given the inequalities in vulnerability and exposure to PM_2.5_ previously discussed, as well as poor access to health services, specific subpopulations (e.g., low-income groups, females, and rural residents) may inequitably suffer from more PM_2.5_-related health burden. Identifying theses inequalities in PM_2.5_-health associations is crucial for understanding the mechanism of PM_2.5_-related health effects, as well as maximizing the outcomes of air pollution control and public health intervention. However, uncertainties and inconsistencies still exist as mentioned above. Therefore, more studies are needed to provide further evidence on the vulnerabilities and the exposures to PM_2.5_ of specific subpopulations in Asia-Pacific for establishing a solid ground where policymakers and public health practitioners are able to prevent or eliminate health inequities caused by the inequalities in PM_2.5_-health associations.

## Conclusion

In Asia-Pacific, previous cohort studies had covered extensive health effects of long-term exposure to ambient PM_2.5_, although they were only conducted in Australia, Mainland China, Hong Kong, Taiwan, and Korea. Consistent evidence was reported and supporting that long-term exposure to ambient PM_2.5_ increased all-cause/non-accidental and CVD mortality as well as the incidences of CVD, T2DM, kidney diseases, and COPD, though research on other outcomes was inconsistent or inadequate. More evidence is required to identify the inequalities in PM_2.5_-health associations for preventing or eliminating potential health inequities. Several research gaps are identified for future studies, including the health effects of long-term exposure to ambient PM_2.5_ in understudies countries and subpopulations, the associations of long-term exposure to ambient PM_2.5_ with the mortalities of diabetes mellitus, kidney diseases, dementia, and lung cancers, as well as issues in study designs, especially the exposure assessment methods.

## Supplementary information

ESM 1 (DOCX 393 kb)

## Data Availability

All data and material used in this study are publicly available.
