# Supplementary material for: Health Effects of Long-Term Exposure to Ambient PM2.5 in Asia-Pacific: a Systematic Review of Cohort Studies
Source: Curr Environ Health Rep. 2022 Mar 16;9(2):130–51. doi: 10.1007/s40572-022-00344-w (PMC9090712; doi:10.1007/s40572-022-00344-w)
Supplement: Supplementary file 1 — (DOCX 393 kb) [file 40572_2022_344_MOESM1_ESM.docx]

**Section 1.** PRISMA Checklist

| **Section/topic** | **#** | **Checklist item** | **Reported on page #** |
| --- | --- | --- | --- |
| **TITLE** | | |  |
| Title | 1 | Identify the report as a systematic review, meta-analysis, or both. | 1 |
| **ABSTRACT** | | |  |
| Structured summary | 2 | Provide a structured summary including, as applicable: background; objectives; data sources; study eligibility criteria, participants, and interventions; study appraisal and synthesis methods; results; limitations; conclusions and implications of key findings; systematic review registration number. | 3 |
| **INTRODUCTION** | | |  |
| Rationale | 3 | Describe the rationale for the review in the context of what is already known. | 4 |
| Objectives | 4 | Provide an explicit statement of questions being addressed with reference to participants, interventions, comparisons, outcomes, and study design (PICOS). | 4 |
| **METHODS** | | |  |
| Protocol and registration | 5 | Indicate if a review protocol exists, if and where it can be accessed (e.g., Web address), and, if available, provide registration information including registration number. | 4-5 |
| Eligibility criteria | 6 | Specify study characteristics (e.g., PICOS, length of follow-up) and report characteristics (e.g., years considered, language, publication status) used as criteria for eligibility, giving rationale. | 5-6 |
| Information sources | 7 | Describe all information sources (e.g., databases with dates of coverage, contact with study authors to identify additional studies) in the search and date last searched. | 5 |
| Search | 8 | Present full electronic search strategy for at least one database, including any limits used, such that it could be repeated. | 5 |
| Study selection | 9 | State the process for selecting studies (i.e., screening, eligibility, included in systematic review, and, if applicable, included in the meta-analysis). | 5-6 |
| Data collection process | 10 | Describe method of data extraction from reports (e.g., piloted forms, independently, in duplicate) and any processes for obtaining and confirming data from investigators. | 6 |
| Data items | 11 | List and define all variables for which data were sought (e.g., PICOS, funding sources) and any assumptions and simplifications made. | 6-7 |
| Risk of bias in individual studies | 12 | Describe methods used for assessing risk of bias of individual studies (including specification of whether this was done at the study or outcome level), and how this information is to be used in any data synthesis. | 6 |
| Summary measures | 13 | State the principal summary measures (e.g., risk ratio, difference in means). | 6-7 |
| Synthesis of results | 14 | Describe the methods of handling data and combining results of studies, if done, including measures of consistency (e.g., I^2^) for each meta-analysis. | 6-7 |
| **Section/topic** | **#** | **Checklist item** | **Reported on page #** |
| Risk of bias across studies | 15 | Specify any assessment of risk of bias that may affect the cumulative evidence (e.g., publication bias, selective reporting within studies). |  |
| Additional analyses | 16 | Describe methods of additional analyses (e.g., sensitivity or subgroup analyses, meta-regression), if done, indicating which were pre-specified. |  |
| **RESULTS** | | |  |
| Study selection | 17 | Give numbers of studies screened, assessed for eligibility, and included in the review, with reasons for exclusions at each stage, ideally with a flow diagram. | 7 |
| Study characteristics | 18 | For each study, present characteristics for which data were extracted (e.g., study size, PICOS, follow-up period) and provide the citations. | 7 |
| Risk of bias within studies | 19 | Present data on risk of bias of each study and, if available, any outcome level assessment (see item 12). | 7 |
| Results of individual studies | 20 | For all outcomes considered (benefits or harms), present, for each study: (a) simple summary data for each intervention group (b) effect estimates and confidence intervals, ideally with a forest plot. | 8-12 |
| Synthesis of results | 21 | Present results of each meta-analysis done, including confidence intervals and measures of consistency. |  |
| Risk of bias across studies | 22 | Present results of any assessment of risk of bias across studies (see Item 15). |  |
| Additional analysis | 23 | Give results of additional analyses, if done (e.g., sensitivity or subgroup analyses, meta-regression [see Item 16]). |  |
| **DISCUSSION** | | |  |
| Summary of evidence | 24 | Summarize the main findings including the strength of evidence for each main outcome; consider their relevance to key groups (e.g., healthcare providers, users, and policy makers). | 12-15 |
| Limitations | 25 | Discuss limitations at study and outcome level (e.g., risk of bias), and at review-level (e.g., incomplete retrieval of identified research, reporting bias). | 15 |
| Conclusions | 26 | Provide a general interpretation of the results in the context of other evidence, and implications for future research. | 18 |
| **FUNDING** | | |  |
| Funding | 27 | Describe sources of funding for the systematic review and other support (e.g., supply of data); role of funders for the systematic review. | 1 |

**Section 2.** Search Strategies in major Chinese literature databases.

Database: China National Knowledge Infrastructure

**
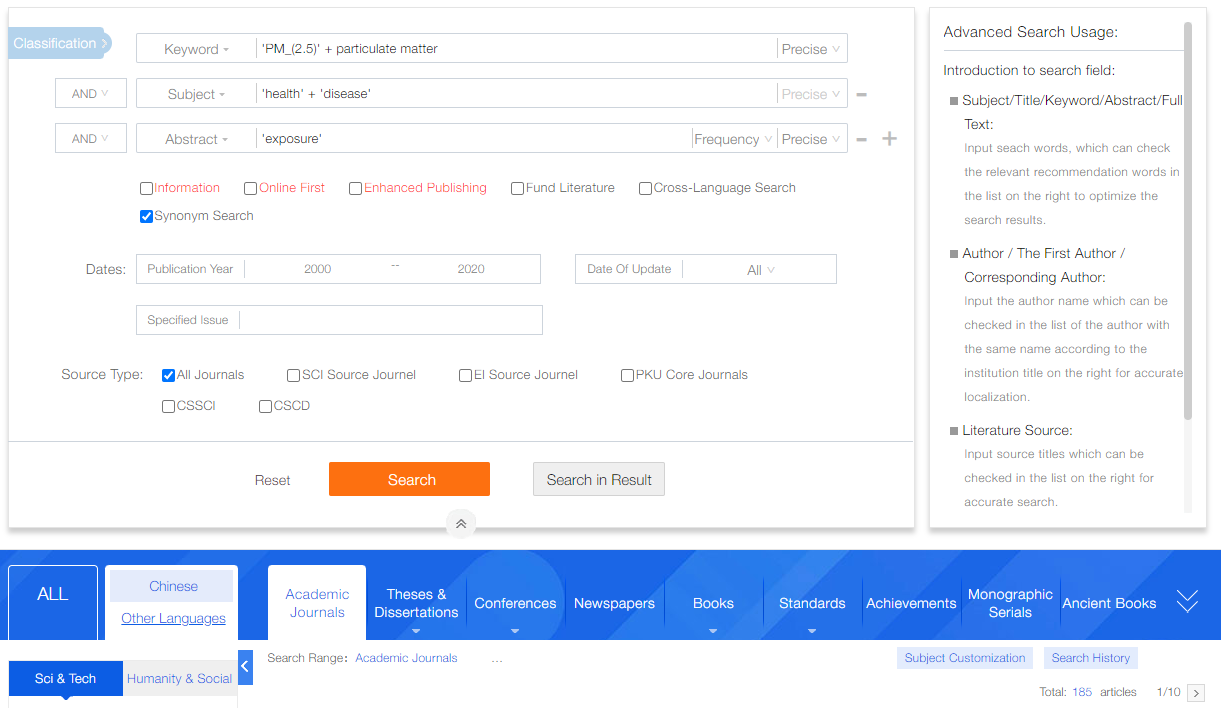
**

Database: VIP Information

**
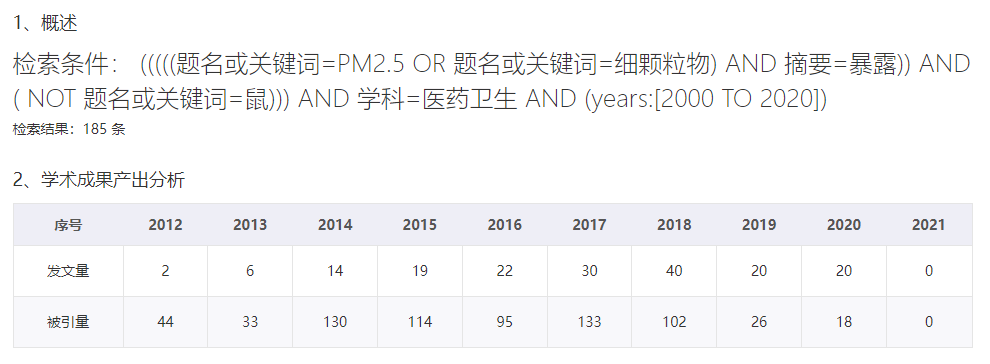
**

**Section 3.** Search Strategies in Medline, Embase, and Web of Science.

Database(s): **Ovid MEDLINE(R) and Epub Ahead of Print, In-Process, In-Data-Review & Other Non-Indexed Citations, Daily and Versions(R)**1946 to July 02, 2021
Search Strategy:

| **#** | **Searches** | **Results** |
| --- | --- | --- |
| 1 | ((air adj1 pollut*) or air quality or (atmospher* adj1 pollut*) or (air adj1 contamina*) or particulate matter* or fine particle*).mp. | 128135 |
| 2 | (cohort stud* or cohort analys* or ((follow up or follow-up or followup) adj1 stud*) or longitudinal stud* or prospective stud* or retrospective stud*).mp. | 2401980 |
| 3 | cross over studies/ or cross-sectional studies/ or case control studies/ or Seroepidemiologic Studies/ or exp clinical trial/ or case reports/ | 3758930 |
| 4 | exp africa/ or exp americas/ or exp europe/ or exp middle east/ or exp kazakhstan/ or exp Kyrgyzstan/ or exp Tajikistan/ or exp Turkmenistan/ or exp Uzbekistan/ or exp antarctic regions/ or exp arctic regions/ | 3592390 |
| 5 | (1 and 2) not (3 or 4) | 2588 |
| 6 | limit 5 to yr="2000 - 2020" | 2001 |

Database(s): **Embase Classic+Embase**1947 to 2021 July 02
Search Strategy:

| **#** | **Searches** | **Results** |
| --- | --- | --- |
| 1 | ((air adj1 pollut*) or air quality or (atmospher* adj1 pollut*) or (air adj1 contamina*) or particulate matter* or fine particle*).mp. | 182321 |
| 2 | (cohort stud* or cohort analys* or ((follow up or follow-up or followup) adj1 stud*) or longitudinal stud* or prospective stud* or retrospective stud*).mp. | 2531046 |
| 3 | cross over studies/ or cross-sectional studies/ or exp case control studies/ or exp clinical trial/ or exp in vivo study/ or exp model/ or exp theoretical study/ | 16155835 |
| 4 | exp africa/ or exp caspian sea/ or exp europe/ or exp western hemisphere/ or exp "arctic and antarctic"/ | 3877534 |
| 5 | (1 and 2) not (3 or 4) | 870 |
| 6 | limit 5 to yr="2000 - 2020" | 714 |

Database(s): **Web of Science**, **-**July 6, 2021


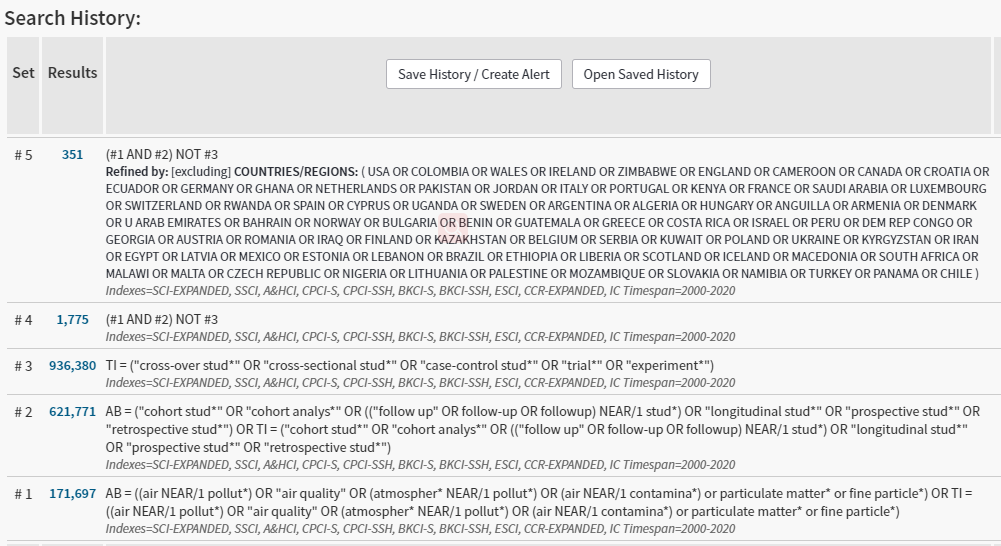


**Table S1.** Newcastle-Ottawa quality assessment scale.

| Authors, year | Selection | | | |  | Comparability |  | Outcome | | | Score |
| --- | --- | --- | --- | --- | --- | --- | --- | --- | --- | --- | --- |
|  | 1 | 2 | 3 | 4 |  | 1 |  | 1 | 2 | 3 |  |
| Bo et al., 2018 | - | - | * | * |  | ** |  | * | * | * | 7 |
| Chan et al., 2018 | - | - | * | * |  | ** |  | * | * | * | 7 |
| Chang et al., 2016 | * | * | - | * |  | ** |  | * | * | * | 8 |
| Chen et al., 2019 | * | * | * | * |  | ** |  | * | * | * | 9 |
| Chen et al., 2020 | - | - | * | * |  | ** |  | * | * | - | 6 |
| Chin et al., 2018 | * | * | * | - |  | * |  | * | * | * | 8 |
| Fan et al., 2018 | * | * | - | * |  | ** |  | * | * | * | 8 |
| Guo et al., 2018 | - | - | * | * |  | ** |  | * | * | * | 7 |
| Guo et al., 2020a | - | - | * | * |  | ** |  | * | * | * | 7 |
| Guo et al., 2020b | - | - | * | * |  | ** |  | * | * | * | 7 |
| Han et al., 2020 | * | * | * | * |  | ** |  | * | * | * | 9 |
| Hanigan et al., 2019 | * | * | * | * |  | ** |  | * | - | * | 8 |
| Hendryx et al., 2019 | * | * | - | * |  | ** |  | - | * | * | 7 |
| Hong et al., 2020 | * | * | - | * |  | ** |  | - | * | * | 7 |
| Huang et al., 2014 | - | - | - | * |  | ** |  | * | * | * | 6 |
| Huang et al., 2019a | * | * | * | * |  | ** |  | * | * | * | 9 |
| Huang et al., 2019b | * | * | * | * |  | ** |  | * | * | * | 9 |
| Hwang et al., 2015 | - | - | * | * |  | ** |  | * | * | * | 7 |
| Jung et al., 2015 | * | * | - | * |  | ** |  | * | * | * | 8 |
| Jung et al., 2019a | * | * | * | * |  | ** |  | * | * | * | 9 |
| Jung et al., 2019b | * | * | * | * |  | ** |  | * | * | * | 9 |
| Kim et al., 2016 | * | * | - | * |  | ** |  | * | * | * | 8 |
| Kim et al., 2017 | * | * | - | * |  | ** |  | * | * | * | 8 |
| Kim et al., 2019 | * | * | - | * |  | ** |  | * | * | * | 8 |
| Kim et al., 2020a | * | * | - | * |  | ** |  | * | * | * | 8 |
| Kim et al., 2020b | * | * | - | * |  | ** |  | * | * | * | 8 |
| Lai et al., 2016 | - | - | - | * |  | ** |  | * | * | * | 6 |
| Lao et al., 2019 | - | - | * | * |  | ** |  | * | * | * | 7 |
| Lee et al., 2019 | * | * | * | * |  | ** |  | * | * | * | 9 |
| Li et al., 2018 | * | * | * | * |  | ** |  | * | * | * | 9 |
| Li et al., 2019 | * | * | * | * |  | ** |  | * | * | * | 9 |
| Li et al., 2020a | * | * | * | * |  | ** |  | * | * | * | 9 |
| Li et al., 2020b | * | * | * | * |  | ** |  | * | * | * | 9 |
| Liang et al., 2019 | * | * | * | * |  | ** |  | * | * | - | 8 |
| Liang et al., 2020 | * | * | * | * |  | ** |  | * | * | * | 9 |
| Lin et al., 2018 | * | * | - | * |  | ** |  | * | * | * | 8 |
| Lin et al., 2019 | * | * | * | * |  | ** |  | * | * | - | 8 |
| Lin et al., 2020a | * | * | * | * |  | ** |  | * | * | * | 9 |
| Lin et al., 2020b | * | * | * | * |  | ** |  | * | * | * | 9 |
| Lv et al., 2020 | * | * | * | * |  | ** |  | * | * | - | 8 |
| Noh et al., 2019 | * | * | - | * |  | ** |  | * | * | * | 8 |
| Norback et al., 2019 | - | - | * | * |  | ** |  | - | * | * | 6 |
| Pan et al., 2016 | - | - | * | * |  | ** |  | * | * | * | 7 |
| Peng et al., 2017 | * | * | * | * |  | ** |  | * | * | * | 9 |
| Qiu et al., 2017 | - | - | * | * |  | ** |  | * | * | * | 7 |
| Qiu et al., 2018 | - | - | * | * |  | ** |  | * | * | * | 7 |
| Ran et al., 2020a | - | - | * | * |  | ** |  | * | * | - | 6 |
| Ran et al., 2020b | - | - | - | * |  | ** |  | * | * | - | 5 |
| Salimi et al., 2018 | - | - | * | * |  | ** |  | * | * | * | 7 |
| Shin et al., 2020a | * | * | - | * |  | ** |  | * | * | * | 8 |
| Shin et al., 2020b | * | * | - | * |  | ** |  | * | * | * | 8 |
| Sun et al., 2020 | - | - | * | * |  | ** |  | * | * | * | 7 |
| Tseng et al., 2015 | - | - | - | * |  | ** |  | * | * | * | 6 |
| Wang et al., 2020 | * | * | * | * |  | ** |  | * | * | * | 9 |
| Wei et al., 2019 | * | * | - | * |  | ** |  | * | * | * | 8 |
| Yang et al., 2018 | - | - | * | * |  | ** |  | * | * | * | 7 |
| Yang et al., 2020 | * | * | * | * |  | ** |  | * | * | * | 9 |
| Yin et al., 2017 | * | * | * | * |  | ** |  | * | * | * | 9 |
| Zhang et al., 2019 | - | - | * | * |  | ** |  | * | * | - | 6 |
| Zhang et al., 2020 | - | - | * | * |  | ** |  | * | * | * | 7 |

Table S2. Health effect estimates not plotted in forest plots.

| **Reference** | **Country/region** | **Population** | **Sample size** | **Outcome** | **Health effect estimates** |
| --- | --- | --- | --- | --- | --- |
| Bo et al., 2019 | Taiwan | General population | 66,702 | Dyslipidemia incidence | 1^st^ tertile: reference  2^nd^ tertile: HR = 1.02 (0.98,1.06)  3^rd^ tertile: HR = 1.08 (1.04,1.13) |
| Chang et al., 2016 | Taiwan | General population | 244,413 | Rheumatoid arthritis incidence | 1^st^ quartile: reference  2^nd^ quartile: HR = 1.22 (0.85, 1.74)  3^rd^ quartile: HR = 1.15 (0.82, 1.62)  4^th^ quartile: HR = 0.79 (0.53, 1.16) |
| Chen et al., 2020 | Taiwan | Elderly population | 360 | Cognitive impairment incidence | 1^st^ tertile: reference  2^nd^ tertile: HR = 2.23 (0.76, 6.55)  3^rd^ tertile: HR = 4.56 (1.51, 13.82) |
| Chin et al., 2018 | Taiwan | T2DM patients | 812 | ACR annual change, per 10 µg/m^3^ | 0.09 mg/g |
| Fan et al., 2018 | Taiwan | General population | 162,797 | Nasopharyngeal carcinoma incidence | 1^st^ quartile: reference  2^nd^ quartile: HR = 2.01 (1.16, 3.49)  3^rd^ quartile: HR = 1.38 (0.78, 2.45)  4^th^ quartile: HR = 1.97 (1.13, 3.43) |
| Hendryx et al., 2019 | Australia | Women | 31,362 for COPD; 29,064 for asthma | COPD or asthma incidence, per unit (log kilograms) | COPD: HR = 1.024 (1.007, 1.041)  Asthma: HR = 1.004 (0.985-1.023) |
| Hong et al., 2020 | Taiwan | Children | 218,008 | Recurrent headache incidence | 1^st^ quartile: reference  2^nd^ quartile: HR = 1.29 (1.25, 1.34)  3^rd^ quartile: HR = 1.57 (1.51, 1.62)  4^th^ quartile: HR = 1.75 (1.69, 1.81) |
| Huang et al., 2014 | Taiwan | Patients undergoing PD | 175 | Dialysis-related infection incidence | 1^st^ half: reference  2^nd^ half: HR = 2.0 (1.03-3.91) |
| Hwang et al., 2015 | Taiwan | Children | 2941 | FVC change, per 10 µg/m^3^ | -42.9 (-67.0, -18.4) ml in boys  -35.7 (-55.0, -16.4) ml in girls |
|  |  |  |  | FEV1 change, per 10 µg/m^3^ | -47.5 (-70.7, -24.3) ml in boys  -31.7 (-52.0, -11.6) ml in girls |
|  |  |  |  | FEF_25-27_ change, per 10 µg/m^3^ | -65.0 (-113.3, -16.7) ml in boys  -28.0 (-78.2, 22.2) ml in girls |
| Jung et al., 2015 | Taiwan | Elderly people | 95,690 | Alzheimer’s Disease incidence | HR = 1.008 (0.946, 1.067) per 10 µg/m^3^ increase of exposure |
| Lao et al., 2019 | Taiwan | General population | 147,908 | T2DM incidence | 1^st^ quartile: reference  2^nd^ quartile: HR = 1.28 (1.18, 1.39)  3^rd^ quartile: HR = 1.27 (1.17, 1.38)  4^th^ quartile: HR = 1.16 (1.07, 1.26) |
| Lin et al., 2018 | Taiwan | General population | 161,970 | Nephrotic Syndrome incidence | 1^st^ quartile: reference  2^nd^ quartile: HR = 1.33 (0.91, 1.93)  3^rd^ quartile: HR = 1.96 (1.08, 3.55)  4^th^ quartile: HR = 2.53 (1.08, 5.94) |
| Lin et al., 2019 | Taiwan | Women | 91,803 | Polycystic Ovary Syndrome incidence | 1^st^ quartile: reference  2^nd^ quartile: HR = 0.26 (0.19, 0.36)  3^rd^ quartile: HR = 3.94 (3.39, 4.58)  4^th^ quartile: HR = 3.56 (3.05, 4.15) |
| Li et al., 2020a | China mainland | General population | 118,551 | Lung cancer incidence | 1^st^ quintile: reference  2^nd^ quintile: HR = 1.44 (1.10, 1.88)  3^rd^ quintile: HR = 1.49 (1.12, 1.99)  4^th^ quintile: HR = 2.08 (1.42, 3.04)  5^th^ quintile: HR = 2.45 (1.83, 3.29) |
|  |  |  |  | Lung cancer mortality | 1^st^ quintile: reference  2^nd^ quintile: HR = 1.83 (1.33, 2.50)  3^rd^ quintile: HR = 1.80 (1.29, 2.53)  4^th^ quintile: HR = 2.50 (1.62, 3.86)  5^th^ quintile: HR = 2.95 (2.09, 4.17) |
| Shin et al., 2020a | South Korea | General population | 115,728 | Senile cataract | 1^st^ quartile: reference  2^nd^ quartile: HR = 1.01 (0.83, 1.23)  3^rd^ quartile: HR = 0.93 (0.76, 1.13)  4^th^ quartile: HR = 0.91 (0.77, 1.06) |
| Shin et al., 2020b | South Korea | General population | 85,869 | Fasting blood glucose change in 2 years | 1^st^ quartile: 0.55 (0.28, 0.82)  2^nd^ quartile: 0.98 (0.70, 1.25)  3^rd^ quartile: 0.69 (0.42, 0.95)  4^th^ quartile: 1.18 (0.89, 1.48) |
|  |  |  |  | Total cholesterol change in 2 years | 1^st^ quartile: 0.19 (-0.23, 0.62)  2^nd^ quartile: 0.46 (0.02, 0.90)  3^rd^ quartile: 0.08 (−0.35, 0.50)  4^th^ quartile: 0.70 (0.23, 1.17) |
|  |  |  |  | Triglycerides change in 2 years | 1^st^ quartile: −0.40 (−1.43, 0.63)  2^nd^ quartile: −0.52 (−1.58, 0.53)  3^rd^ quartile: − 0.24 (−1.26, 0.79)  4^th^ quartile: −0.37 (− 1.51, 0.77) |
|  |  |  |  | High-density lipoprotein cholesterol change in 2 years | 1^st^ quartile: − 0.22 (−0.42, 0.02)  2^nd^ quartile: 0.03 (− 0.18, 0.23)  3^rd^ quartile: − 0.21 (− 0.40, 0.01)  4^th^ quartile: − 0.21 (−0.43, 0.01) |
|  |  |  |  | Low-density lipoprotein cholesterol change in 2 years | 1^st^ quartile: 0.34 (− 0.09, 0.77)  2^nd^ quartile: 0.62 (0.18, 1.06)  3^rd^ quartile: 0.32 (− 0.11, 0.74)  4^th^ quartile: 1.38 (0.91, 1.86) |
| Wei et al., 2019 | Taiwan | Children | 97,306 | Myopia incidence | 1^st^ quartile: reference  2^nd^ quartile: HR = 0.84 (0.80, 0.89)  3^rd^ quartile: HR = 1.69 (1.61, 1.76)  4^th^ quartile: HR = 1.76 (1.68, 1.83) |
